# Matrix Metalloproteinase-9 Production by Immortalized Human Chondrocyte Lines

**DOI:** 10.4172/2155-9899.1000422

**Published:** 2016-06-03

**Authors:** Charles J. Malemud, Evan C. Meszaros, Meredith A. Wylie, Wissam Dahoud, Yelenna Skomorovska-Prokvolit, Sam Mesiano

**Affiliations:** 1 Department of Medicine, Division of Rheumatic Diseases, Case Western Reserve University, School of Medicine, Cleveland, Ohio 44106, USA; 2 Department of Reproductive Biology, Case Western Reserve University, School of Medicine, Cleveland, Ohio 44106, USA; 3 Department of Pathology, Institute of Pathology, Case Western Reserve University, School of Medicine, University Hospitals Case Medical Center, Cleveland, Ohio 44106, USA

**Keywords:** Chondrocytes, Human, Matrix metalloproteinases, Osteoarthritis, Pro-inflammatory cytokines, Rheumatoid arthritis, Signal transduction

## Abstract

We reported at the Keynote Forum of Immunology Summit-2015 that recombinant human (rh) TNF-α or rhIL-6 stimulated production of matrix metalloproteinase-9 (MMP-9) in the T/C28a2 and C-28/I2 human immortalized chondrocyte cell lines. Furthermore, we reported that tocilizumab (TCZ), a fully humanized monoclonal antibody which neutralizes IL-6-mediated signaling, inhibited the rhIL-6-mediated increase in the production of MMP-9. IL-6 is also a known activator of the JAK/STAT signaling pathway. In that regard, we evaluated the effect of rhIL-6 on total and phosphorylated Signal Transducer and Activator of Transcription by these chondrocyte lines which showed that whereas STAT3 was constitutively phosphorylated in T/C28a2 chondrocytes, rhIL-6 activated STAT3 in C-28/I2 chondrocytes. The finding that rhIL-6 increased the production of MMP-9 by human immortalized chondrocyte cell lines may have important implications with respect to the destruction of articular cartilage in rheumatoid arthritis and osteoarthritis. Thus, the markedly elevated level of IL-6 in rheumatoid arthritis and osteoarthritis sera and synovial fluid would be expected to generate significant MMP-9 to cause the degradation of articular cartilage extracellular matrix proteins. The finding that TCZ suppressed rhIL-6-mediated MMP-9 production suggests that TCZ, currently employed in the medical therapy of rheumatoid arthritis, could be considered as a drug for osteoarthritis.

## Introduction

Although it is acknowledged that the pathologic mechanisms that contribute to rheumatoid arthritis (RA) and osteoarthritis (OA) are significantly different, there are overlapping pathophysiological components which are characteristic of each disease process [1]. Most prominent of these overlapping characteristics of OA and RA is the significant increase in the serum and synovial fluid levels of pro-inflammatory cytokines, exemplified by tumor necrosis factor-α (TNF-α), interleukin-1β (IL-1β) and IL-6 [2,3]. Thus, presumably acting in concert these pro-inflammatory cytokines can cause 1) matrix metalloproteinase (MMP) gene expression to be up-regulated; 2) decreased synthesis of extracellular matrix (ECM) proteins, such as Type II collagen and aggrecan; and 3) induction of chondrocyte apoptosis [1].

The pro-inflammatory cytokines, IL-6 and TNF-α, are considered most integral in perpetuating the inflammatory response in RA and OA [2,3]. IL-6 and TNF-α also play a role in up-regulating MMP gene expression as well [4-6]. In that regard, the MMPs most often associated with chronic inflammation characteristic of RA and OA are MMP-1 (collagenase-1), MMP-2 (72kDa gelatinase), MMP-3 (stromelysin-1), MMP-10 (stromelysin-2), and MMP-9 (92kDa gelatinase) [6]. MMP-9 appears to be particularly critical for the progression of both RA and OA pathology primarily because MMP-9 possesses broad ECM protein substrate specificity. Thus, MMP-9 is a potent enzyme mediator of cartilage destruction based on the accumulation of evidence which showed that MMP-9 could efficiently degrade many of the articular cartilage ECM proteins that are critical for maintaining the structural integrity of this musculoskeletal tissue [7-10].

Despite recent basic research advances and clinical findings which has vastly improved our understanding of the role of pro-inflammatory cytokines and MMPs in RA and OA disease pathogenesis and progression, a detailed understanding of which mechanism(s) contribute to the capacity of TNF-α or IL-6 to produce these changes in human chondrocyte MMP-9 remains to be elucidated. Thus, the collection of results from experimental studies presented at the Keynote Forum at Immunology Summit-2015 reported on the extent to which recombinant human TNF-α (rhTNF-α) or rhIL-6 altered MMP-9 production by human chondrocyte immortalized cell lines. A second line of evidence presented at Immunology Summit-2015 showed that tocilizumab (TCZ), a fully humanized monoclonal antibody, which neutralizes the activity of IL-6 *via* IL-6Rα/gp130 or by interacting with membrane-bound IL-6R (absent the gp130 component) or with the soluble IL-6 receptor (sIL-6R), reduced MMP-9 production by the immortalized human chondrocyte lines in the presence of recombinant human (rh) IL-6.

## Materials

The materials used in these studies were reviewed at Immunology Summit-2015. Thus, the immortalized human chondrocyte cell lines, T/C28a2 and C-28/I2, were provided by Drs. Mary Goldring and Miguel Otero (The Hospital for Special Surgery, Weill Medical College of Cornell University). These immortalized human chondrocyte lines had previously been shown to synthesize several “signature” ECM proteins of human cartilage [11-13] as well as the cartilage-specific transcription factor, SOX9 [12].

PANC-1, a pancreatic tumor cell line was obtained from the American Type Culture Collection. PANC-1 was incubated with phorbol myristate acetate. PANC-1 was employed as the positive control for MMP-9 production [14]. The pro-inflammatory cytokines, rhIL-6 and rhTNF-α were obtained from various commercial vendors as was sIL-6R as previously described [14]. U0126, a small molecule inhibitor of MEK1/2, an upstream protein kinase required for the phosphorylation of ERK1/2 was purchased from Cell Signaling Technology. The Signal Transducer and Activator of Transcription-3 (U-STAT3) antibodies were purchased from R&D Systems and the β-actin antibody from Cell Signaling Technology [15]. An antibody which interacts with human neutrophil gelatinase-associated lipocalin (NGAL) was purchased from Pierce Biotechnology [14]. WHI-P131 (Janex-1) was from Cayman Chemicals. Tocilizumab (TCZ) was obtained through a contract between Case Western Reserve University and Genentech/Roche Group.

## Methods

We thoroughly reviewed the methodology at Immunology Summit-2015 which was employed for these studies. MMP-9 production was measured by an MMP-9 ELISA using our now published method [14]. MMP-9 production was also assessed by immunocytochemical (ICC) localization of MMP-9 in C-28/I2 chondrocytes [14]. The presence of NGAL was also determined by ICC [14]. In addition, the experimental details for analyzing MMP-9 production as well as for the detection of STAT proteins by western blotting were performed as described in 2 papers published following the Keynote Presentation at Immunology Summit-2015 [14,15].

## Results

We reported at Immunology Summit-2015 that MMP-9 production was significantly increased by rhIL-6 (50ng/ml) or by rhTNF-α (20ng/ml) in both T/C28a2 and C-28/I2 chondrocyte cell lines as well as by PANC-1 cells treated with phorbol myristate acetate. We also noted that MMP-9 production by these chondrocytes after incubation with rhTNF-α was far more robust compared to rhIL-6. We noted that TCZ (200 ng/ml) inhibited rhIL-6-stimulated, but not rhTNF-α-induced MMP-9 production, after 1 and 4 hrs. However, higher concentrations of TCZ (i.e. 400-800 ng/ml) did not appreciably increase the inhibition of MMP-9 when combined with rhIL-6 (50 ng/ml) for 1 or 4 h [14]. The ICC analysis confirmed the MMP-9 ELISA data. Thus, we reported that in the presence of rhIL-6 the number of MMP-9-positive C-28/I2 chondrocytes was reduced by both TCZ (200 ng/ml) as well as by sIL-6R (50 ng/ml), whereas the combination of rhIL-6 plus sIL-6R significantly increased the number of MMP-9-positive chondrocytes compared to sIL-6R alone [14].

We had previously reported that NGAL existed in a complex with MMP-9 in synovial fluid sampled from OA patients prior to undergoing joint replacement surgery [10]. In addition, we previously showed that the NGAL/MMP-9 complex protected MMP-9 from autodegradation [10]. At Immunology Summit-2015, we reported that the level of immunoreactive NGAL as determined by ICC was decreased after treatment of C-28/I2 chondrocytes with sIL-6R (50 ng/ml) or after incubation with the combination of rhIL-6 (50 ng/ml) plus TCZ (200 ng/ml) when compared to a “no-additions” control group, but not when compared to rhIL-6 alone. Of note, the combination of rhIL-6 plus TCZ did not reduce NGAL-positive chondrocytes compared to rhIL-6 alone.

We also showed western blots at Immunology summit-2015 demonstrating that STAT1 was constitutively phosphorylated in the T/C28a2 chondrocyte line [16]. However, C-28/I2 chondrocytes incubated with rhIL-6 (50 ng/ml) resulted in the phosphorylation of STAT3 without changing total STAT3 [15]. Moreover, the combination of rhIL-6 and the pan-JAK-small molecule inhibitor. Janex-1 (100 μM), resulted in the complete loss of the anti-p-STAT3 antibody reactive band [15]. In addition, western blot analyses shown at Immunology Summit-2015 indicated that besides total STAT3 (U-STAT3) which migrated to a position similar to authentic U-STAT3 (i.e. 75 kDa) often termed STAT3α as previously reported [17], a slightly faster migrating anti-STAT3 antibody reactive band was also detected [15].

At the Immunology Summit-2015 Keynote presentation we proposed that although IL-6 was known to preferentially activate the JAK/STAT pathway [17], IL-6 could also potentially activate components of the SAPK/MAPK pathway as well. To examine this possibility we showed that C-28/I2 chondrocytes produced 2 isoforms of phosphorylated-p38kinase which were identified on western blots with a p-p38kinase-α antibody. These p-p38kinase-α isoforms were p-p38kinase-55kDa and p-p38kinase-38kDa [18]. However, treatment of C-28/I2 chondrocytes with rhIL-6 for 30 min did not alter the signal intensity of either of the p-p38kinase-α isoforms. Of note, the combination of rhIL-6 (50 ng/ml) and U0126 (10 μM or 20 μM) reduced the signal intensity of both p-p38kinase isoforms [18].

## Discussion

To summarize the results of these *in vitro* studies presented at Immunology Summit-2015 we showed that TCZ, a monoclonal antibody that neutralizes the activity of IL-6 mainly *via* IL-6/IL-6Rα/gp130 signaling, inhibited rhIL-6-stimulated MMP-9 production in the immortalized human chondrocyte cell lines, T/C28a2 and C-28/I2, whereas the combination of rhTNF-α and TCZ did not inhibit MMP-9 production [14]. Thus, the results of these *in vitro* analyses may have important implications for the future therapy of OA for suppressing MMP-9 production by articular chondrocytes.

Furthermore, sIL-6R also reduced the number of MMP-9-positive C-28/I2 chondrocytes in the presence of rhIL-6 [14]. Thus, in addition to rhIL-6-mediated activation of the IL-6/IL-6Rα/gp130 and IL-6/mIL-6R pathways we proposed at Immunology Summit-2015 that a signaling pathway activated when IL-6 interacts with sIL-6R was also likely to be involved in IL-6-mediated chondrocyte MMP-9 production. Furthermore, based on this data as well as other evidence published from our laboratory [15], we proposed that neutralizing the IL-6-mediated stimulation of chondrocyte MMP-9 with TCZ or administration of an exogenous sIL-6R fusion protein could ultimately become another medical strategy for suppressing articular cartilage ECM protein degradation in OA or RA.

We had previously shown using a quantitative immunoblot analysis that rhIL-6, rhTNF-α or sIL-6R activated STAT3 protein in human immortalized chondrocyte cell lines [15,16]. We had also shown that incubating human chondrocytes enzymatically liberated from OA knee cartilage with rhTNF-α (10 ng/ml) resulted in the phosphorylation of U-STAT3 (p-STAT3) without altering total U-STAT3α [19]. This data also provided direct evidence that rhTNF-α (which is not commonly associated with activation of JAK/STAT signaling), could activate U-STAT3 in these immortalized human chondrocytes as well as in cultured human OA chondrocytes.

The results of western blots also indicated that additional STAT protein isoforms were produced by these human chondrocyte cell lines. These STAT protein isoforms reacted with anti-U-STAT or anti-p-STAT antibodies and migrated faster than authentic U-STAT proteins [15]. However, at the very least we confirmed in that study [15] that these faster migrating STAT isoforms did not result from proteolysis during the processing of chondrocyte protein lysates employed for these western blots.

It may also be noteworthy in the present context that the faster migrating STAT proteins may turn out to be truncated STAT proteins which were previously found to be produced by myeloid leukemia cells where a truncated isoform of STAT3 (e.g. STAT3β) [20] was eventually traced to the activity of a serine-dependent protease produced by these cells acting on STAT3α [21]. Following that discovery, a protease-mediated truncation of the COOH-terminal transactivation domain of STAT3α was found to be the molecular mechanism responsible for the truncated STAT3β isoform [22]. More recently, Timofeeva et al. [23] reported the existence of a 67.5 kDa truncated U-STAT3 isoform which appeared to recognize single-stranded spacers within cruciform DNA structures. This truncated STAT3 isoform was abundant in the nuclei of cancer cells and due to this finding Timofeeva et al. [23] also suggested that the truncated U-STAT3 isoform could play a role in the organization of chromatin and gene expression in these cells. However, presently we do not know if any relationship exists between the faster migrating isoform of U-STAT3 produced by the C-28/I2 immortalized human chondrocyte cell line and any of the truncated form(s) of U-STAT3 and p-STAT3 produced by cancer cells. This will in all likelihood require further in-depth analysis going forward to determine the extent to which the faster migrating STAT3 isoform produced by this human immortalized chondrocyte cell line arises from a post-synthesis enzymatic processing of U-STAT3α or by synthesis from alternatively spliced U-STAT3α mRNA.
